# Cytokine-Coding Oncolytic Adenovirus TILT-123 Is Safe, Selective, and Effective as a Single Agent and in Combination with Immune Checkpoint Inhibitor Anti-PD-1

**DOI:** 10.3390/cells10020246

**Published:** 2021-01-27

**Authors:** Riikka Havunen, Riikka Kalliokoski, Mikko Siurala, Suvi Sorsa, João M. Santos, Victor Cervera-Carrascon, Marjukka Anttila, Akseli Hemminki

**Affiliations:** 1 TILT Biotherapeutics Ltd., 00290 Helsinki, Finland; riikka.havunen@helsinki.fi (R.H.); riikka.kalliokoski@tiltbio.com (R.K.); mikko.siurala@pennmedicine.upenn.edu (M.S.); suvi@tiltbio.com (S.S.); joao@tiltbio.com (J.M.S.); victor@tiltbio.com (V.C.-C.); 2 Cancer Gene Therapy Group, Translational Immunology Research Program and Department of Oncology, University of Helsinki, 00290 Helsinki, Finland; 3 Pathology Unit, Finnish Food Authority, 00790 Helsinki, Finland; marjukka.anttila@ruokavirasto.fi; 4 Helsinki University Hospital Comprehensive Cancer Center, 00290 Helsinki, Finland

**Keywords:** adenovirus, oncolytic virus, immunotherapy, safety, biodistribution

## Abstract

Oncolytic viruses provide a biologically multi-faceted treatment option for patients who cannot be cured with currently available treatment options. We constructed an oncolytic adenovirus, TILT-123, to support T-cell therapies and immune checkpoint inhibitors in solid tumors. Adenoviruses are immunogenic by nature, are easy to produce in large quantities, and can carry relatively large transgenes. They are the most commonly used gene therapy vectors and are well tolerated in patients. TILT-123 expresses two potent cytokines, tumor necrosis factor alpha and interleukin-2, to stimulate especially the T-cell compartment in the tumor microenvironment. Before entering clinical studies, the safety and biodistribution of TILT-123 was studied in Syrian hamsters and in mice. The results show that TILT-123 is safe in animals as monotherapy and in combination with an immune checkpoint inhibitor anti-PD-1. The virus treatment induces acute changes in circulating immune cell compartments, but the levels return to normal by the middle of the treatment period. The virus is rapidly cleared from healthy tissues, and it does not cause damage to vital organs. The results support the initiation of a phase 1 dose-escalation trial, where melanoma patients receiving a tumor-infiltrating lymphocyte therapy are treated with TILT-123 (NCT04217473).

## 1. Introduction

Oncolytic adenovirus therapy was approved for the treatment of advanced head and neck cancer in China in combination with chemotherapy in 2005 [1]. Since then, several different approaches aiming to improve treatment efficacy have been taken in clinical phase 1 and 2 trials [2,3,4]. Typically, new oncolytic adenovirus product candidates carry one or two transgenes coding for immunostimulatory agents, such as GM-CSF, CD40L, or 4-1BBL [2,5]. These approaches are further studied in combination with other treatments, such as immune checkpoint inhibitors [6].

Oncolytic adenovirus treatments have generally been well tolerated in humans. The typical adverse events are grades 1 and 2, including flu-like symptoms, headache, nausea, fatigue and dizziness, anemia, leuko- and thrombocytopenia, elevated liver transaminases, and diarrhea. Before using a new therapy on patients, it is important to evaluate the safety in preclinical models. Syrian hamsters serve as a good preclinical model for adenovirus product safety because it is semi-permissive for adenovirus replication, unlike mice, which are nonpermissive [7]. Immunocompromised mice with human xenografts can, however, provide complementary data on the direct effects of an oncolytic virus or the transgenes on healthy tissues.

Here, we investigate the preclinical safety and biodistribution of an oncolytic adenovirus TILT-123 (Ad5/3-E2F-d24-hTNFa-IRES-hIL2). TILT-123 is a chimeric adenovirus based on type 5 with a fiber knob from type 3, which is used to increase the tumor cell transfection through desmoglein-2 [8,9]. The E2F promoter and the 24-base-pair (bp) deletion in constant region 2 of E1A restrict viral replication to cancer cells defective for the p16-retinoblastoma pathway. The virus codes for two transgenes: human Tumor Necrosis Factor alpha (TNFa) and Interleukin-2 (IL-2). TILT-123 was designed to enable and enhance T-cell therapies and immune checkpoint inhibitors in solid tumors [10]. It has shown complete responses in animals treated in combination with tumor-infiltrating lymphocytes, Chimeric Antigen Receptor (CAR) T cells, or immune checkpoint inhibitors anti-PD-1 and anti-PD-L1 [10,11,12,13]. Also, the use of TILT-123 has been shown to remove the need for preconditioning chemotherapy and postconditioning IL-2, typically used in adoptive cell therapies [14,15]. The results of this study support the initiation of a phase 1 trial, investigating the combination of TILT-123 and tumor-infiltrating lymphocyte therapy in melanoma (NCT04217473).

## 2. Materials and Methods

### 2.1. Animals and Treatments

**Figure 1:**
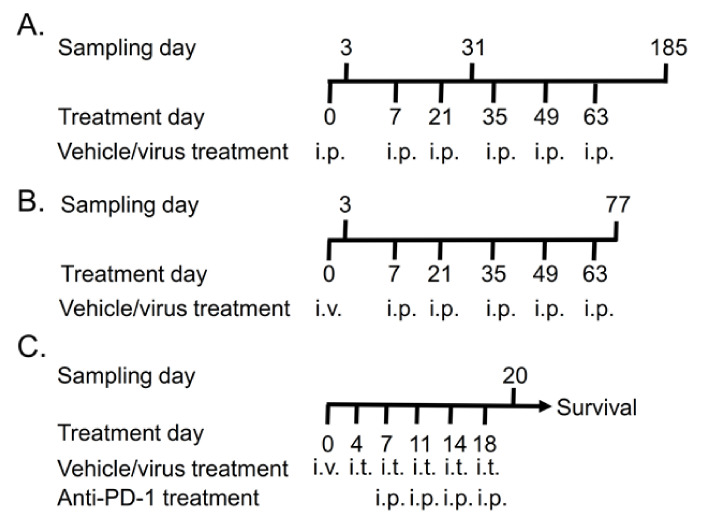
Treatment schedule for TILT-123 safety studies: (**A**) 15 male and female hamsters received saline or 1 × 10^10^, 1 × 10^11^, or 1 × 10^12^ virus particles (VP)/kg of TILT-123 six times intraperitoneally (i.p.). Five animals/sex/group were sacrificed 3, 31, and 185 days after the first treatment. (**B**) Ten male and female hamsters received saline, 1 × 10^11^ VP/kg, or 1 × 10^12^ VP/kg of TILT-123 once intravenously (i.v.) and five times i.p. Five animals/sex/group were sacrificed 3 days after the first injection and two weeks after the last injections (on day 77). (**C**) Human fibrosarcoma tumors were implanted subcutaneously to both flanks of nude NMRI mice. Nine days after implantation, the animals received 1 × 10^5^ VP, 1 × 10^7^ VP, or 1 × 10^9^ VP i.v. The following treatments were injected intratumorally (i.t.) Anti-PD-1 (100 µg) treatments were started on day 7 after two virus treatments. Five animals per group were sacrificed on day 20, and the rest (6–7 animals/group) continued in the survival study.

A biodistribution and toxicity study was conducted in Syrian hamsters following Good Laboratory Practices (GLP) at Cellvax S.A.S. (Paris, France). Syrian hamsters, aged 6–7 weeks males and females, were purchased from Janvier Labs (Le Genest-Saint-Isle, France) and acclimatized for at least three days before the treatments. The animals received six TILT-123 injections in total (days 0, 7, 21, 35, 49, and 63). The first injection was either intravenous (i.v., retro-orbital) or intraperitoneal (i.p.). The other injections were given i.p. The doses were diluted with 0.9% sodium chloride (sterile, i.v. quality, Laboratoires Gilbert), which was also administered to the mock control animals (Figure 1A,B).

Hamster wellbeing was followed daily, and weight development was recorded once a week. In addition, food and water consumption was recorded weekly. On predetermined days, five male and female animals per group were sacrificed to collect blood, secretions (saliva swab, urine, and feces), and organs (brain, kidneys, ovaries, testes, epididymis, liver, prostate, uterus, heart, lungs, spleen, and vagina) for biodistribution analyses, histopathology, blood cell analysis, and serum chemistry analyses.

Nude/NMRI female mice (Harlan Laboratories, Indianapolis, IN, USA) were acclimatized for seven days before experimentation. Human fibrosarcoma tumors (HT-1080) were engrafted subcutaneously and allowed to grow until 0.5 mm in diameter. The animals received 1 × 10^5^ virus particles (VP), 1 × 10^7^ VP, or 1 × 10^9^ VP (corresponding to approximately 4 × 10^6^–4 × 10^10^ VP/kg) twice a week, six times in total (Figure 1C). The first injection was given i.v. into the tail vein, and the following injections were given intratumorally (i.t.). In addition, one group was treated with the combination of 1 × 10^9^ VP and 100 µg of murine anti-PD-1 (InVivoMAb anti-mouse PD-1 (CD279), BioXcell, Lebanon, NH, USA). The control groups received saline (PBS) or anti-PD-1 alone. Anti-PD-1 administration started after two virus treatments and was given i.p. Five animals per group were sacrificed on day 21 to collect samples (blood, heart, lung, liver, kidney, spleen, and tumor) for virus biodistribution, toxicity, and cytokine analyses. The rest 6–7 animals/group were left for survival experiment, where tumor growth and animal weight development were followed.

### 2.2. Biodistribution

TILT-123 distribution in animals was evaluated by qPCR. A real-time TaqMan^®^ PCR method for TILT-123 detection and quantification in hamster tissues and biofluids was developed and validated at Accelero Bioanalycs GmbH (Berlin, Germany). The primers for detecting the virus genomes in hamster tissues were designed to amplify a sequence between IRES and IL-2 in TILT-123, a region which does not appear in any naturally occurring naïve specimens: forward primer CGAACCACGGGGACGTGGT; reverse primer GCAAGACTTAGTGCAATGCAAGACAGG; probe FAM-GATAATATGGCCACAACCATGTACAGG-MGB. Before qPCR, an external PCR was first used to amplify the target sequence (forward primer GCACATGCTTTACATGTGTTTAGTCGAGGT; reverse primer GAAGTAGGTGCACTGTTTGTGACAAGTGC). The hamster GAPDH housekeeping gene was analyzed as a control [16]. The limit for detection was 100 VP/sample (in 200 µL), and the limit for quantification was between 500 and 2 × 10^7^ VP/sample.

Mouse tissues were analyzed by detecting the 24-bp deletion region from adenovirus E1A. Beta-acting gene expression served as a reaction control [17].

### 2.3. Histopathology

The collected hamster organs, except for epididymitis and testis, were fixed in 4% formalin for at least 48 h. The epididymitis and testes were fixed in Davidson’s fluid for 48 h, then washed, and stored in 70% ethanol at room temperature before further processing. Mouse tissues were fixed with 10% formalin for 48 h and stored in 70% ethanol. The tissues were embedded in paraffin wax, cut to 4-μm sections, and stained with hemalum, or hematoxylin and eosin. Veterinary pathologists analyzed and scored the slides.

### 2.4. Blood Analyses

Blood was collected by heart puncture from anesthetized or euthanized animals. Blood cells and hemoglobin parameters were analyzed from hamster blood treated with the K2EDTA anticoagulant. Coagulation parameters and blood chemistry were analyzed from hamster plasma separated from the whole blood in citrate tubes or in lithium heparin, respectively. Mouse blood was collected into dry tubes and the serum was separated by centrifugation after blood clotting.

### 2.5. Neutralizing Antibodies

Ad5/3-neutralizing antibodies were analyzed from hamster serums 3 days after i.v. injection and two weeks after the treatment period (day 77) as described before [18]. Briefly, A549 cells were infected with luciferase-expressing Ad5/3-Luc1 virus [19] mixed with heat-treated serum samples at different dilutions (four-fold dilution series from 1:4 to 1:16384). A standard dilution series was formed from rabbit serum containing Ad5/3 neutralizing antibodies. Luciferase expression was measured with Luciferase Assay System kit (Promega, Madison, WI, USA). The neutralizing antibody titer was determined as the lowest degree of dilution that blocked gene transfer by >80%, as compared with the assay condition where Ad5/3-Luc1 was not neutralized with an antiserum.

### 2.6. Transgene Expression

The expression of TILT-123 transgenes human TNFa and human IL-2 were analyzed from hamster and mouse serums with human ELISA kits (Abcam, Cambridge, UK or R&D Systems, Minneapolis, MN, USA). The serum was extracted from whole blood in dry tubes. In addition, the transgenes were analyzed from homogenized mouse organs and tumors. The cytokine levels in tissues were normalized against the total protein content in the sample, measured with a Bradford assay.

### 2.7. Selective Replication In Vitro

Selective replication of TILT-123 was studied in A549 lung adenocarcinoma cells (positive control, purchased from ATCC, Manassas, VA, USA), human primary hepatocytes (Lonza Verviers SPRL, Bruxelles, Belgium), human MRC-5 fibroblasts (ATCC), and human vascular endothelial HUVEC cells (Millipore). The cells were plated on 24-well plates and infected with TILT-123, wild type Adenovirus 5 (VR-1516, ATCC), Ad5/3 replicative control virus (a similar selection device as TILT-123 but no cytokine payload), or Ad5/3-Luc1 non-replicative control virus [10,19]. Infected cells and cell culture supernatants were harvested 72 h post-infection by centrifugation.

The virus was released from the cells by repeating freeze–thaw cycles four times in total. DNA was extracted from the samples with the phenol/chloroform/isoamyl alcohol (25:24:1) method and the virus copy numbers determined by detecting the adenovirus E4 copy number in samples by qPCR [20]. Infectious virus particles were determined with TCID50 assay, where a sample dilution series was plated on A549 cells and cytopathic effect formation was followed for ten days. TILT-123 transgene production was measured from the cell culture supernatants with ELISA, as described above.

### 2.8. Statistical Analysis

Statistical analyses were performed with GraphPad Pism 8.2 using the Mann–Whitney or ANOVA test. Tumor growth curves, animal weight development, and water and food consumption were analyzed using linear mixed model analysis in IBM SPSS Statistics version 24.0.0.2. A *p*-value smaller than 0.05 was considered significant.

## 3. Results

### 3.1. Repeated TILT-123 Injections Do Not Cause Visual Signs of Organ Damage in Hamsters

Syrian hamsters were injected six times i.p. or once i.v. and five times i.p. with TILT-123 to investigate virus biodistribution and toxicity in a replication-permissive immunocompetent model. The doses (low, mid, and high) corresponded to the ones planned to be used in a clinical setting, ranging from 1 × 10^10^ VP/kg to 1 × 10^12^ VP/kg. Repeated TILT-123 injections did not cause premature deaths or visual signs of organ damage in hamsters. All hamsters had positive weight development over time, but the treatment period caused temporary weight loss (Figure S1). The treated hamsters consumed slightly less food but more water than the control animals, both of which are typical stress reactions. Hamster weight development, and food and water consumption were similar for the animals that received all injections i.p. and for those that were treated first i.v. and five times i.p.

### 3.2. TILT-123 Induces Acute Immune Reactions in Hamsters

**Figure 2:**
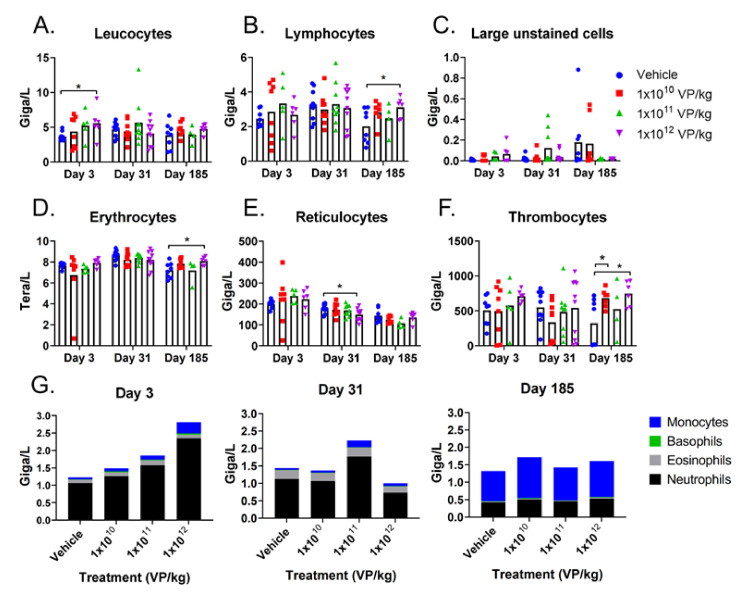
Circulating blood cell levels in hamsters: the hamsters received six TILT-123 injections i.p. at three different doses. Leucocytes (**A**), lymphocytes (**B**), large unstained cells (**C**), red blood cells (**D**), reticulocytes (**E**), thrombocytes (**F**), and myeloblast-derived cells (monocytes, basophils, eosinophils, and neutrophils) (**G**) were counted from the whole blood at three time points (3, 31, and 185 days after the first treatment). The data show the means plus standard error of mean. Statistical significance between the cell number in control group vs. treatment group on each time point was evaluated with the Mann–Whitney test (* *p* < 0.05).

The highest treatment dose increased the number of leucocytes in hamster blood only after the first virus injection (Figure 2A). This was seen especially as an increase in neutrophil levels, which was the dominating myeloblast-derived cell population at an early sampling point and in the middle of the treatment period (Figure 2G). At the last sampling point (six months after the beginning of the study), the dominating myeloblast-derived cell population was monocytes (Figure 2G). Treatments did not influence large unstained cell levels (Figure 2C).

In the middle of the treatment period, the only statistically significant difference in the blood cell populations was a slightly reduced reticulocyte level in animals treated with the highest dose (Figure 2E). Lymphocyte, erythrocyte, and thrombocyte levels were slightly elevated with the highest dose at the last sampling point, and thrombocytes elevated also with the lowest dose (Figure 2B,D,F). However, individual animal-level raw data reveal that half of the control samples had low thrombocyte numbers (<15 Giga/L) whereas the other half had numbers comparable to previous sampling points (>500 Giga/L).

When the first injection was given i.v., the changes in blood cells were similar as when the injection was i.p. (Figure S3). The leucocyte levels were slightly elevated, reflecting elevated lymphocyte, neutrophil, monocyte, and basophil levels. In addition, treatment increased the reticulocyte levels slightly, which was also seen as slightly higher erythrocyte levels. Treated animals had also a higher number of thrombocytes after a single i.v. injection than the control animals, but the treatment did not affect the mean platelet volume (Figure S4). Two weeks after the treatments stopped, the blood cell levels were comparable between the treatment and control groups.

Blood clotting was delayed in some animals in both the treatment and the control groups at the last sampling time point (day 185), when the animals received six i.p. injections (Figure S2H,I). Increased activated, partial thromboplastin time was also seen in samples collected two weeks after the treatment period from animals that received the first injection i.v. and the rest i.p. (Figure S4). Prothrombin time, however, was similar between the groups at both time points. Considering the scattered thrombocyte counts and the phenomenon seen in both treated and control animals, a technical handling error in sample preparation cannot be ruled out. As these findings did not clearly correlate with treatment, they do not raise an obvious safety concern, but blood clotting is a parameter that will, nevertheless, be closely monitored in the clinical phase.

Even though the erythrocyte level was slightly elevated with the highest dose at the last sampling point after i.p. injections, it did not affect the red distribution width (Figure S2B). However, hematocrit, mean corpuscular hemoglobin, and mean corpuscular hemoglobin concentration were also slightly elevated in animals treated with the lowest and highest doses, at the last sampling point (Figure S2C,E,F). Hemoglobin and hematocrit levels were slightly elevated also after i.v. injection, but changes were not observed in other parameters evaluating erythrocyte performance (Figure S4). Taken together, the data suggests that treatment did not significantly affect erythrocytes or their performance.

### 3.3. TILT-123 Does Not Damage Hamster Organs

The serum chemistry analyses provided data for TILT-123 toxicity evaluation together with histopathological analyses. A veterinary pathologist did not observe any treatment-related abnormalities in healthy hamster tissues. Also, no major differences were observed in the serum chemistry values between the vehicle control and treatment groups.

**Figure 3:**
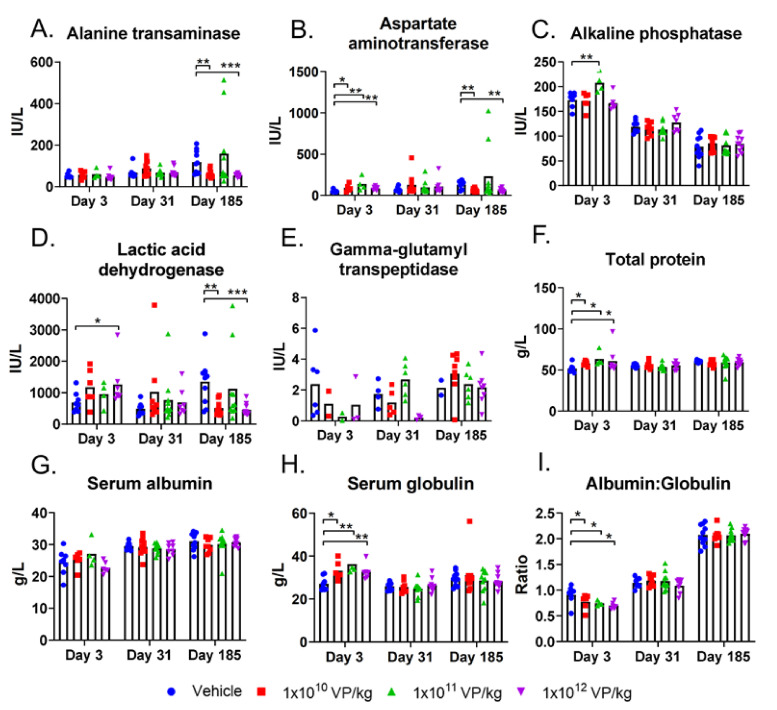
Blood chemistry values in hamsters: the hamsters received six i.p. TILT-123 injections at three different doses. Alanine transaminase (**A**), aspartate aminotransferase (**B**), alkaline phosphatase (**C**), lactic acid dehydrogenase (**D**), gamma-glutamyl transpeptidase (**E**), total protein level (**F**), serum albumin (**G**), and serum globulin (**H**) were measured from hamster serum samples at three time points (3, 31, and 185 days after the first treatment). In addition, albumin to globulin ratio was calculated to depict the relative amounts of these proteins in serum (**I**). The data show means plus standard error of mean. Statistical significance between the molecule levels in the control group vs. treatment group on each time point was evaluated with the Mann–Whitney test (* *p* < 0.05; ** *p* < 0.01; *** *p* < 0.001).

Compared to the control group, i.p. treatment with any dose acutely elevated aspartate aminotransferase and total protein levels in blood (Figure 3B,F, respectively). Of note, sample hemolysis, which was observed in some of the samples, can induce falsely high liver enzyme and lactate dehydrogenase levels. The elevation in total protein level was due to an elevated serum globulin level, which also led to a lower albumin-to-globulin ratio (Figure 3H,I, respectively). At later time points, these parameters were comparable to the control group values.

Middle and high doses also elevated the alkaline phosphatase and lactic acid dehydrogenase levels, respectively, on day 3. All blood values in the treatment groups were comparable to the control group by the middle of the treatment period. At the last sampling time point, day 185, the alanine transaminase, aspartate aminotransferase, and lactic acid dehydrogenase levels were lower in animals treated with the lowest and highest doses compared to the control group (Figure 3A,B,D, respectively).

I.v. injection did not cause worse changes in blood chemistry values than i.p. injections. Alanine transaminase, aspartate aminotransferase, lactic acid dehydrogenase, total protein, and albumin levels were lower in treated animals than in control animals on day 3 (Figure S5). Alkaline phosphatase levels were slightly elevated in treated animals on day 3 but on the same level as the control group values two weeks after the treatments (day 77) (Figure S5C). Like alkaline phosphatase levels, all other serum chemistry values were comparable to the control group values two weeks after treatment, except serum protein and albumin levels, which were slightly elevated in the treatment groups but still close to the control animal values (Figure S5F,G).

**Figure 4:**
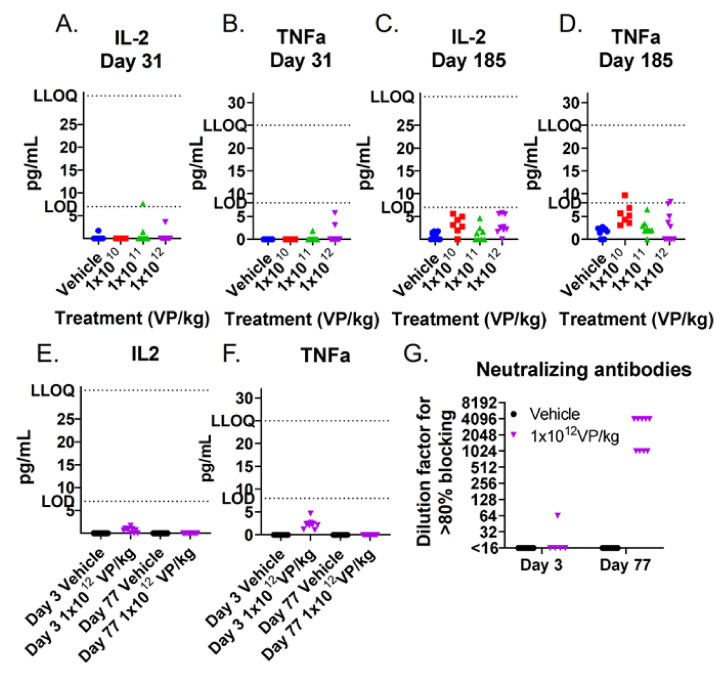
TILT-123 transgene expression and neutralizing antibodies in hamster sera: human TNFa and IL-2 were analyzed from hamster sera by ELISA after three i.p. injections on day 31 (**A**,**B**) and after six injections at the end of the experiment (day 185) (**C**,**D**). In addition, the transgene expression (**E**,**F**) and Ad5/3 neutralizing antibodies (**G**) were measured after one i.v. injection (day 3) and two weeks after the last i.p. injection (day 77). LOD: Limit of Detection; LLOQ: Lower Limit of Quantification.

TILT-123 transgenes human TNFa and IL-2 were also measured from hamster blood at different time points. The levels remained mainly under the method’s lower limit of detection, which indicates that TILT-123 was not replicating in hamsters that were not bearing tumors (Figure 4A–F). This is expected as the virus is designed to replicate in a tumor-specific manner.

### 3.4. TILT-123 Is Rapidly Cleared from Healthy Hamster Tissues

TILT-123 administration led to the formation of neutralizing antibodies during the treatment period (Figure 4G). In one animal, a slight increase in antibody levels was observed already 3 days after the first treatment. All samples were clearly positive two weeks after the entire treatment regimen (day 77). The neutralizing antibodies participated in virus clearance, which occurred in non-tumor-bearing hamsters by the middle of the treatment period.

**Figure 5:**
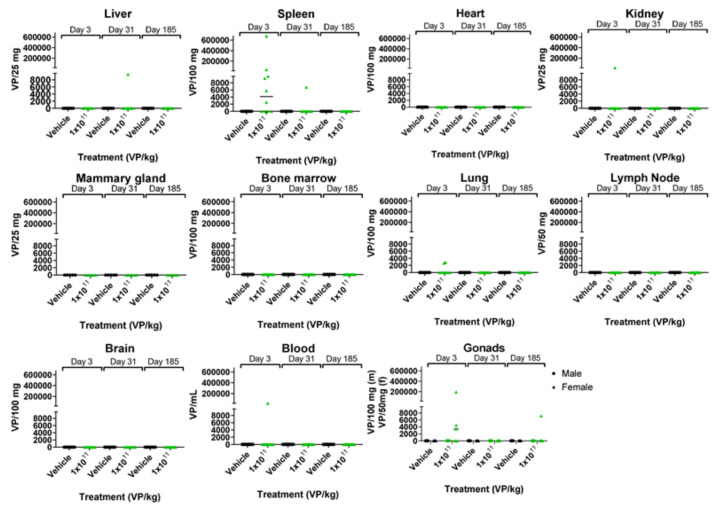
TILT-123 biodistribution in hamsters: the hamsters received six TILT-123 injections i.p. at the dose of 1 × 10^11^ VP/kg. The presence of TILT-123 in hamster tissues was studied with qPCR at three time points (3, 31, and 185 days after the first treatment). Only values above the limit of quantification are shown.

When TILT-123 was administered i.p., virus genomes were mainly found in the spleen and in individual kidney, lung, blood, and gonad samples after the first injection (Figure 5). Most of the samples were negative for the virus genomes by the middle of the treatment period. Only one sample (a female gonad) was positive on day 185.

When TILT-123 was administered i.v., most of the virus genomes were found in the liver and spleen after the first injection (Figure S6). Some positive samples were also obtained from the heart, kidney, lungs, and brain. All but two kidney samples and one lung sample were free from virus genomes two weeks after the treatment period (day 77). Neither of the treatment routes resulted in virus shedding though saliva, urine, or feces (Figure S7).

### 3.5. The Combination of TILT-123 and Anti-PD-1 Is Well Tolerated and Has Antitumor Efficacy in a Mouse Xenograft Study

**Figure 6:**
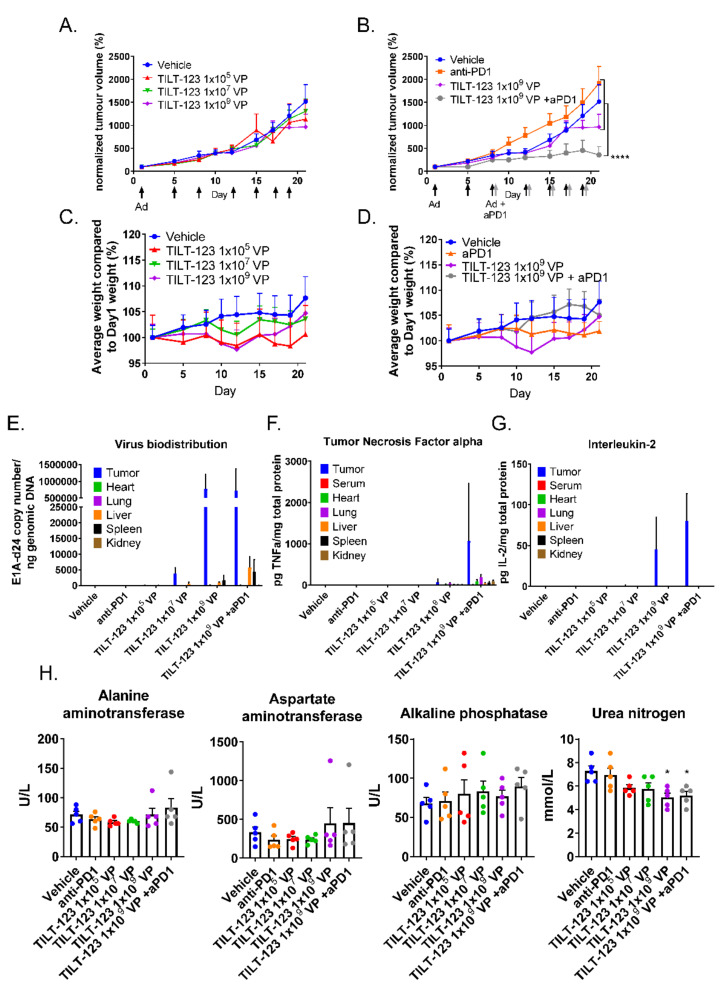
Mice treated with TILT-123 and anti-PD-1 show tumor growth reduction. Nude mice bearing subcutaneous human fibrosarcoma tumors received TILT-123 (black arrows) and anti-PD-1 treatments (grey arrows) twice a week. Anti-PD-1 treatments started on day 7 and were given i.p. The first virus injection was given i.v., and the rest was given i.t. Tumor size was measured with a digital caliper (**A**,**B**), and animals were weighed (**C**,**D**) two to three times a week. Tumor size and animal weight at the beginning of the treatment period was set to 100%. Adenovirus genomes were detected from the tumors and healthy tissue samples (**E**) by qPCR and normalized against the genomic DNA amount or sample volume. TILT-123 transgenes human TNFa (**F**) and human IL-2 (**G**) were analyzed from tumors and healthy tissue samples with ELISA and were normalized to total protein contents in the samples. (**H**) Whole blood was collected from mice 48 h after last treatment (day 20). The serum was separated after blood coagulation. The data show mean plus standard error of mean. Statistical significance was evaluated with the log-linear mixed models analysis (tumor growth and weight development) or with one-way ANOVA. * *p* < 0.05; **** *p* < 0.0001; Ad = TILT-123 injection; aPD1 = anti-PD-1; VP = Virus Particles.

Nude/NMRI mice bearing subcutaneous human fibrosarcoma tumors were treated with TILT-123 monotherapy or in combination with mouse anti-PD-1. Significant tumor growth control was seen with the combination approach (Figure 6A,B). Four out of six animals in the combination group were cured and remained tumor-free until the end of the experiment (day 222). Additionally, one animal was cured with the lowest virus dose (1 × 10^5^ VP) and with anti-PD1 alone. Of note, all cured tumors were among the smallest 25% at the beginning of treatment, indicating that initial size might have influenced the likelihood of a curative response. The control animals gained more weight than the treated animals during the experimental period, but the differences were not statistically significant (Figure 6C,D).

The virus was located mostly in tumors 48 h after the last treatment (day 20) (Figure 6E). Regarding organs, most virus genomes were detected from livers and spleens, similar to the findings from non-tumor-bearing hamsters. Only one serum sample from the TILT-123-treated animals was positive for virus genome copies. Low levels of human TNFa were also found in healthy tissues, but much higher concentrations were present in the tumors (Figure 6F). IL-2 was only detected in tumors (Figure 6G). Treatment with the highest TILT-123 dose with or without anti-PD-1 reduced the serum urea nitrogen levels. All other values were comparable between the control and treatment groups (Figure 6H). Histopathological evaluation of the hearts, spleens, lungs, livers, and kidneys did not reveal treatment-related changes.

### 3.6. TILT-123 Replication Is Restricted to Cancer Cells

**Figure 7:**
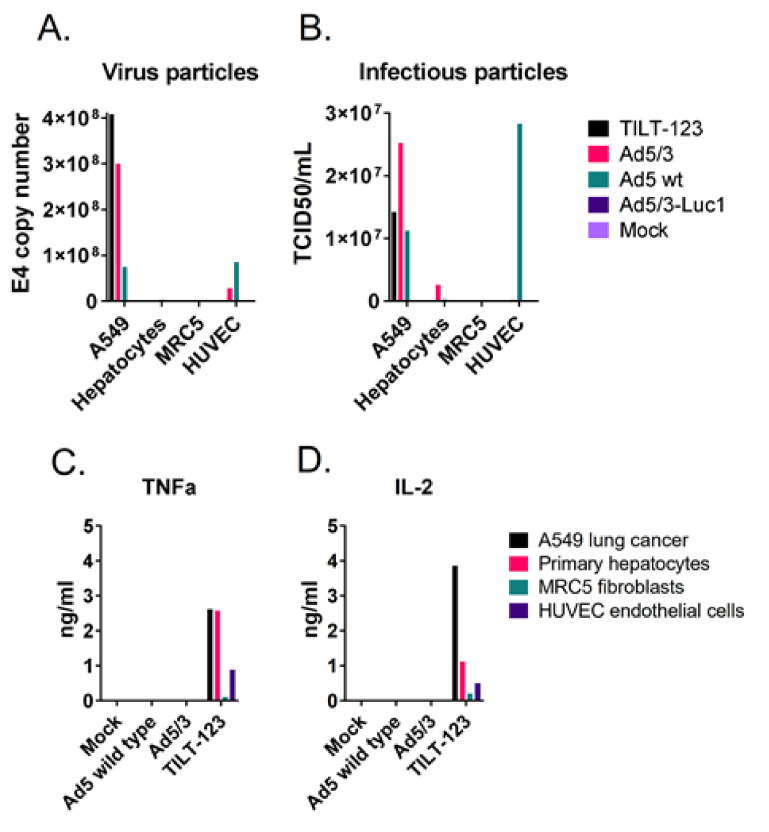
TILT-123 replicates selectively in cancer cells in vitro. A549 lung adenocarcinoma cells, human primary hepatocytes, MRC5 fibroblasts, and human vascular endothelial HUVEC cells were infected with TILT-123, Ad5/3 replicative control virus (similar selection device as TILT-123 but no cytokine payload), wild-type adenovirus type 5 (Ad5 wt), or replication-incompetent Ad5/3-Luc1 virus or left uninfected as a control (mock). (**A**) Virus genomes and (**B**) infectious virus particles were analyzed from the infected cells 72 h after infection. TILT-123 transgenes TNFa (**C**) and IL-2 (**D**) were analyzed from cell culture supernatants.

As some virus was observed in healthy tissues after treatments, selective replication was studied further in vitro. Human primary hepatocytes, vascular endothelial cells, and fibroblasts were infected with TILT-123 or the control viruses, and the presence of physical and infectious virus particles were studied 72 h post-infection. Neither physical nor infectious TILT-123 particles were detected in the normal cells (Figure 7A,B). However, some TNFa and IL-2 were found from the cell culture supernatants, indicating that low levels of transgene expression might occur in these cells in vitro even though functional virus particles are not formed (Figure 7C,D).

## 4. Discussion

New treatment modalities are urgently needed, especially for metastatic and recurrent cancer. Immunotherapies provide promising results in part of this patient population, but complete responders are still rare [21]. Response rates can be improved with combination therapies, for example, when oncolytic viruses are combined with immune checkpoint inhibitors [22]. As opposed to combinations of two checkpoint inhibitors, preliminary data indicate that oncolytic virus with one checkpoint inhibitor does not seem to increase toxicity over single-agent treatment in humans [22]. Preclinical studies on treatment toxicities are, nevertheless, important to perform prior to human trials.

Here, we evaluated the preclinical safety of TILT-123 as a monotherapy and in combination with an immune checkpoint inhibitor anti-PD-1. Treatment doses, schemes, and routes of administration mimic the clinical setting. Monotherapy studies were conducted in Syrian hamsters, which allow human adenovirus replication, including chimeric 5/3 type adenoviruses [7,10]. The combination of TILT-123 and anti-PD-1 was studied in nude mice, as a hamster anti-PD-1 has not been described to date. This model allowed TILT-123 replication in the implanted tumors and transduction of healthy tissues.

Because the hamster study included long-term follow up, it was not feasible to have the animals bear tumors. Thus, all injections were given i.p. To compare different systemic routes of administration, some of the hamsters received the first injection i.v. Because the aim of the mouse study was to collect acute safety data, tumors could be engrafted and treated i.t. after an initial i.v. treatment, as planned in the clinical setting.

The results show that TILT-123 is safe in animals when administered in clinically relevant (and above) doses (1 × 10^10^–1 × 10^12^ VP/kg). The main difference between i.p. and i.v. injection routes was biodistribution: i.v. administration spread the virus more widely in the body. Virus genomes were mainly found from the spleen and gonads after i.p. administration, whereas when given i.v., the gonads were unaffected but most of the virus was located in the liver. The observed i.p. biodistribution profile is compatible with direct transduction of i.p. organs, while i.v. appears to give access to the liver.

Regarding safety parameters, i.p. and i.v. administration caused similar changes in blood cell compartments and serum chemistry. Not surprisingly, the treatment seemed to increase the number of circulating leucocytes, especially lymphocytes and neutrophils, which are typically the first cell types to emerge into the blood upon infection. The neutrophil population was the dominating myeloblast-derived cell type during the treatment period, but after a recovery period, the number of monocytes increased. It is likely that, during the treatment period, monocytes differentiated into dendritic cells and macrophages as a reaction to infection, which lead to a decrease in monocyte numbers. After a recovery period, the number of monocytes was the largest compartment among myeloblast-derived cells.

The replication of TILT-123 is restricted to cancer cells by two genetic modifications: a tumor specific promoter and a partial deletion in one of the early genes. The selectivity seemed to hold well in vitro, as normal human cell cultures were not producing infectious particles. Low levels of transgene expression seemed to occur, which may relate to virus design, as transgene cassettes are in the E3 early region. Rapid clearance of the virus from the animals, the absence of histopathological changes, and low cytokine levels in the hamster serum indicate that the selectivity devices work well also in vivo. These parameters will be important to monitor in the human trial.

Because TILT-123 is designed to enable T-cell therapies and immune checkpoint inhibitors, additional safety analyses were conducted in mice in combination with anti-PD-1. In immunocompetent animals, the combination results in a 100% survival rate [12]. Surprisingly, antitumor efficacy was observed also in immunocompromised nude/NMRI mice, which lack most immune cell compartments. Nevertheless, the combination might have antitumor effects through immunological reactions because the mice still possess functional B cells, dendritic cells, macrophages, and natural killer (NK) cells. Out of these cell compartments, anti-PD-1 could possibly induce the functionality of B cells and NK cells.

In mice, activated NK cells express PD-1 that suppresses cell functionality when bound to PD-L1 [23]. Regarding tumors with impaired MHC-I expression, NK cells are possibly even more important for antitumor immune reactions than T cells. Moreover, PD-1 and PD-L1 blockades are known to enhance the functionality of NK cells against tumors [23]. Mouse NK cells express more PD-1 when infiltrating tumors than those in the spleen, indicating that the tumor microenvironment might induce the expression of PD-1 [23]. Similarly, human NK cells do not typically express PD-1, but ovarian cancer patients have exceptionally high numbers of these cells in blood and ascites [24]. Pesce et al. reported that these cells are less proliferative and express less IL-2 and IL-15 than PD-1-negative NK cells. Thus, stimulation of NK cells with TILT-123 and anti-PD-1 also has clinical relevance.

B cells have a controversial role in regulating antitumor efficacy: regulatory B cells express PD-L1 and suppress T-cell functionality, but some subtypes produce antibodies and cytokines that promote T-cell activity via dendritic cells [25,26]. B cells can also produce granzyme B, which can kill cancer cells directly. As NMRI mice do not have a T-cell compartment, B cells most probably had a minor role in mediating antitumor efficacy. Nevertheless, activated B cells do express PD-1 and the PD-L1/PD-L2 blockade can induce their activity [27]. When the interaction is blocked, these cells can proliferate more, express activation markers, and produce more IL-6 [27]. In addition, antibody production by B cells could in theory stimulate antibody-dependent cellular cytotoxicity mediated by NK cells. Both antiviral and antitumor antibodies have been implicated as potentially relevant in oncolytic immunotherapy [28].

The addition of anti-PD-1 to a TILT-123 treatment regimen did not cause additive toxicity. As in hamsters, these treatments did not cause histological changes in mouse normal tissues or affect serum chemistry values, even though traces of virus genomes were found in all analyzed healthy tissues when the highest dose was used and in some animals treated with the middle dose. However, the virus was mostly present in tumors, where it can replicate. As human adenovirus entry into mouse tissues is restricted, using human CD46 or desmoglein-2 (adenovirus type 3 receptors) transgenic mice might allow greater transduction and can cause more effects on healthy tissues. In the used model, the highest copy number levels in normal tissues were observed in the spleen and liver, indicating a similar biodistribution as in hamsters. The results are also in accordance with the observations of the biodistribution of other oncolytic adenoviruses [16,29,30,31] and previous reports on 5/3 chimeric adenovirus that have the same capsid as TILT-123 [16,20]. Of note, as NMRI mice lack a complete immune system, any virus leaking from the tumor to normal tissues is not cleared as efficiently as from immunocompetent animals. Thus, the virus levels in normal tissues are expected to be even lower in subjects with a functional immune system.

To summarize, similar safety and biodistribution profiles were seen in immunocompetent hamsters and in immunocompromised mice. Good safety results were not compromised when TILT-123 was combined with anti-PD-1. Overall, the data reported here indicate that the virus does not replicate in normal tissues and that it is rapidly cleared from nontumor organs. These data support clinical translation of TILT-123.

## 5. Conclusions

The study shows that repeated administration of TILT-123 is safe in animals as monotherapy and in combination with an immune checkpoint inhibitor anti-PD-1. Acute changes in blood values indicate that the virus induces immunological reactions in immunocompetent animals but that the values return to normal levels after the acute phase. The virus is also rapidly cleared from healthy tissues, where it cannot replicate. Importantly, the treatment did not cause any tissue-level changes in animals.

## Data Availability

Restrictions apply to the availability of these data.

## Supplementary Materials

The following are available online at https://www.mdpi.com/2073-4409/10/2/246/s1, Figure S1: Hamster weight development, and food and water consumption: the hamsters were weighed at least once a week. Water and food consumption were recorded regularly. (A) The hamsters treated six times i.p. weighed less than the control animals (mean plus standard error of mean are shown). They also consumed less food. The animals treated with high and middle doses consumed more water than the control animals. (B) Animals that were treated first i.v. and then i.p. weighed slightly less than the control animals (mean plus standard error of mean are shown). The highest treatment dose induced lowered food intake but higher water intake. Also, the animals treated with the middle dose consumed more water than the control animals. Statistical difference was evaluated with log-ranked mixed models: ** *p* < 0.01; **** *p* < 0.0001. Figure S2: Hamster blood values: mean platelet volume (A), red distribution width (B), hematocrit (C), hemoglobin (D), mean corpuscular hemoglobin (E), mean corpuscular hemoglobin concentration (F), and mean corpuscular volume (G) were measured from whole blood at three time points (3, 31, and 185 days after the first treatment). In addition, blood coagulation was evaluated by measuring activated partial thromboplastin time (H) and prothrombin time (I). The data show means plus standard error of mean. Statistical significance between the cell numbers in the control group vs. treatment group on each time point was evaluated with the Mann–Whitney test (* *p* < 0.05; ** *p* < 0.01). Figure S3: Hamster blood cells after one intravenous injection and five intraperitoneal injections: whole blood was collected through heart puncture after one intravenous injection (day 3) and two weeks after the last injection (day 77). Statistical difference was evaluated with log-ranked mixed models: * *p* < 0.05; *** *p* < 0.001. Figure S4: Hamster blood values after one intravenous injection and five intraperitoneal injections: whole blood was collected through heart puncture after one intravenous injection (day 3) and two weeks after the last injection (day 77). Statistical significance between the cell numbers in the control group vs. treatment group on each time point was evaluated with the Mann–Whitney test (* *p* < 0.05; ** *p* < 0.01). Figure S5: Blood chemistry values in hamsters treated first intravenously followed by five intraperitoneal treatments: alanine transaminase (ALT) (A), aspartate aminotransferase (AST) (B), alkaline phosphatase (ALP) (C), lactic acid dehydrogenase (LDH) (D), gamma-glutamyl transpeptidase (GGT) (E), total protein level (PROT) (F), serum albumin (ALB) (G), and serum globulin (GLOB) (H) were measured from hamster serum samples at two time points (3 days after the first treatment and two weeks after the last treatment on day 77). In addition, the albumin to globulin ratio (A:G) was calculated to depict the relative amounts of these proteins in serum (I). The data show means plus standard error of mean. Statistical significance between the molecule levels in the control group vs. treatment group on each time point was evaluated with the Mann–Whitney test (* *p* < 0.05; ** *p* < 0.01; **** *p* < 0.0001). LLOQ: Lower Limit of Quantification. Figure S6: TILT-123 biodistribution in hamsters treated intravenously and intraperitoneally: the presence of TILT-123 hamster tissues was studied with qPCR three days after the intravenous injection (day 3) and two weeks after the last intraperitoneal injection (day 77). Only values above limit of quantification are shown. Figure S7: Virus shedding after treatments: TILT-123 shedding through saliva, urine, and feces was studied with qPCR at three time points (3, 31, and 185 days after the first treatment) (A–C), three days after one intravenous injection (day 3, D–F), or two weeks after one intravenous injection and five intraperitoneal injections (day 77, D–F). Only values above the limit of quantification are shown.
