# Supplementary figures and images for: Cytokine-Coding Oncolytic Adenovirus TILT-123 Is Safe, Selective, and Effective as a Single Agent and in Combination with Immune Checkpoint Inhibitor Anti-PD-1
Source: Cells. 2021 Jan 27;10(2):246. doi: 10.3390/cells10020246 (PMC7911972; doi:10.3390/cells10020246)

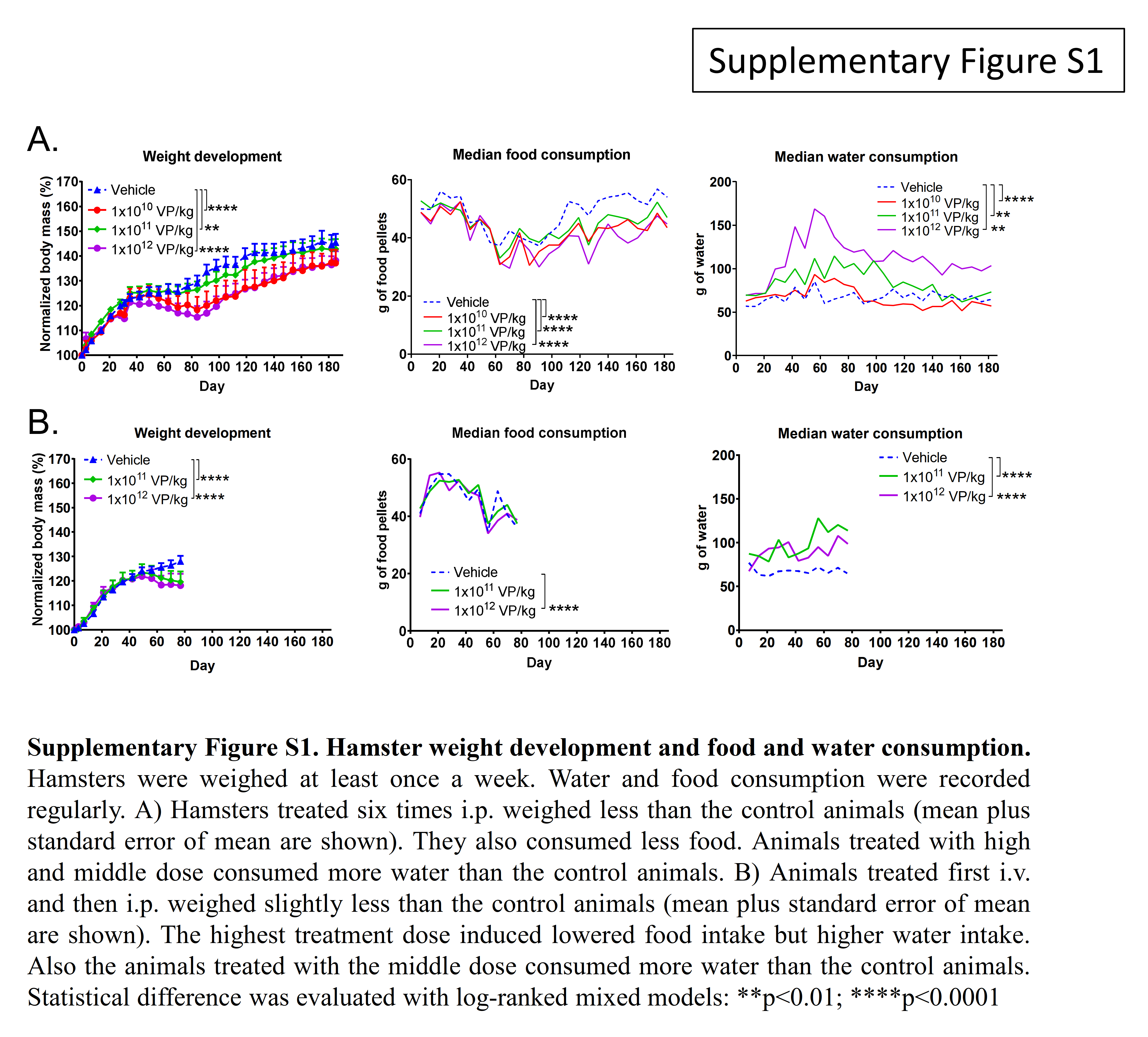

Supplement: Supplementary file 1 [file cells-10-00246-s001.zip › Supplementary figures Havunen 2020/Havunen 2020 Figure_S1.tif]

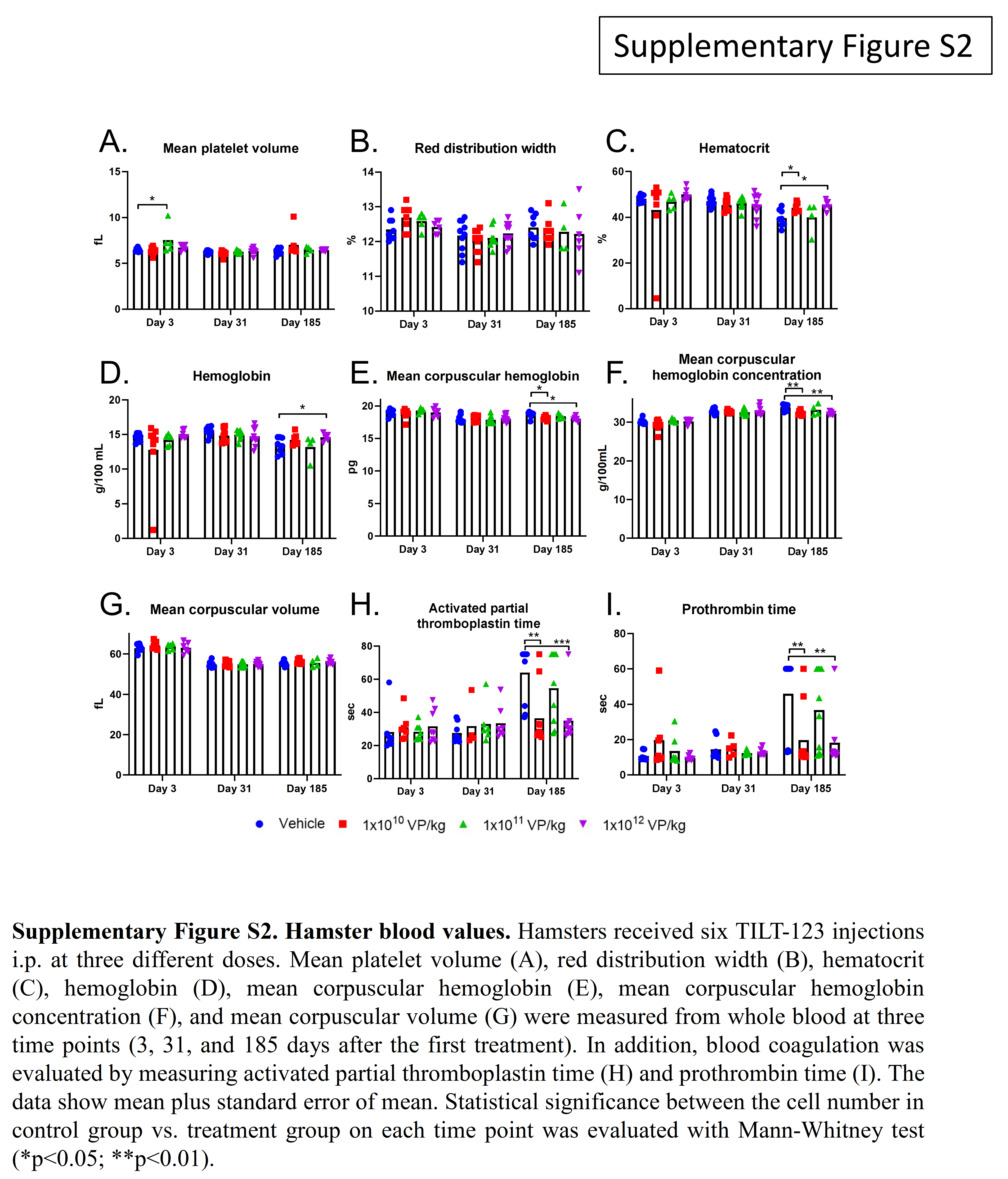

Supplement: Supplementary file 1 [file cells-10-00246-s001.zip › Supplementary figures Havunen 2020/Havunen 2020 Figure_S2.tif]

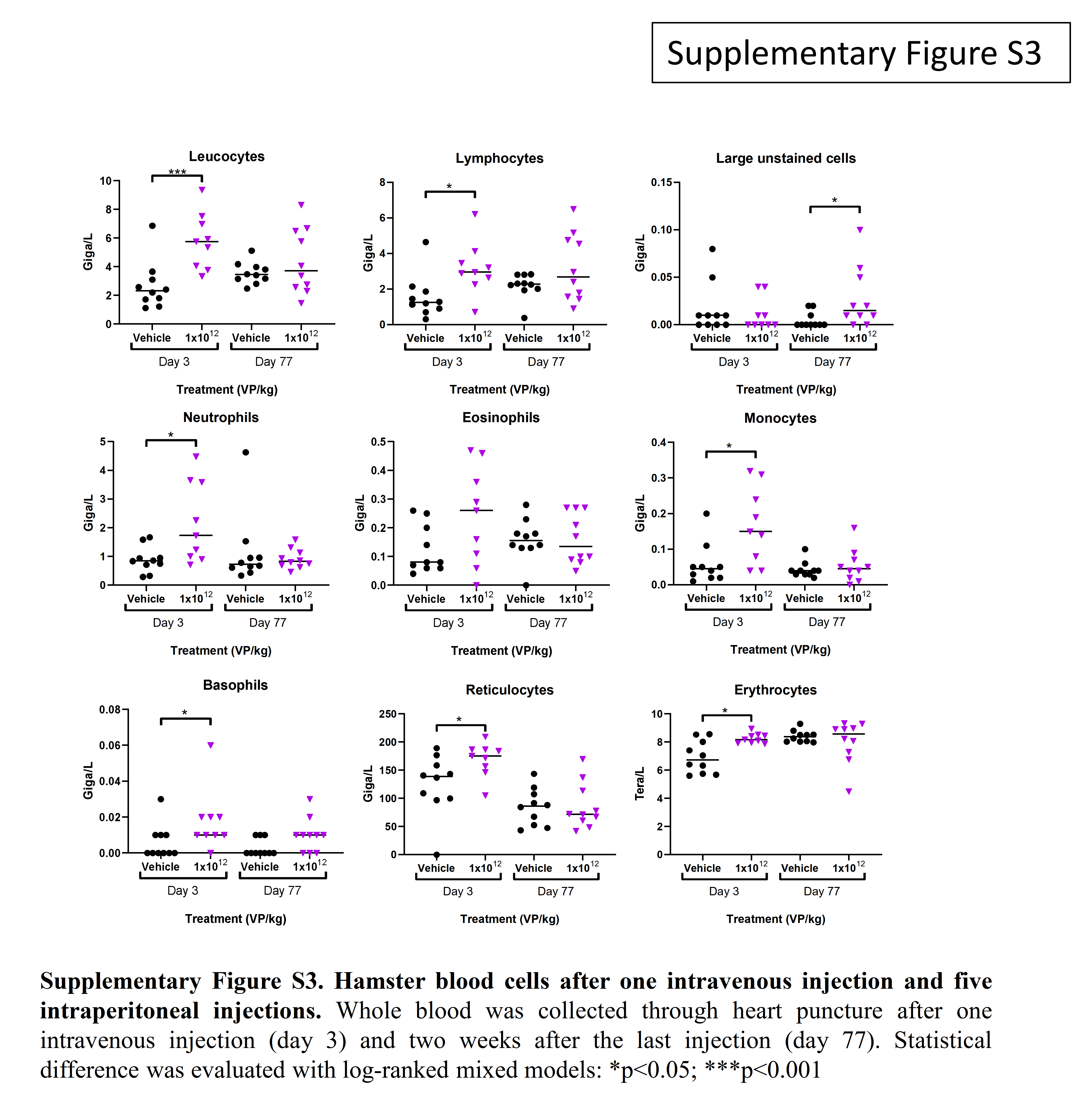

Supplement: Supplementary file 1 [file cells-10-00246-s001.zip › Supplementary figures Havunen 2020/Havunen 2020 Figure_S3.tif]

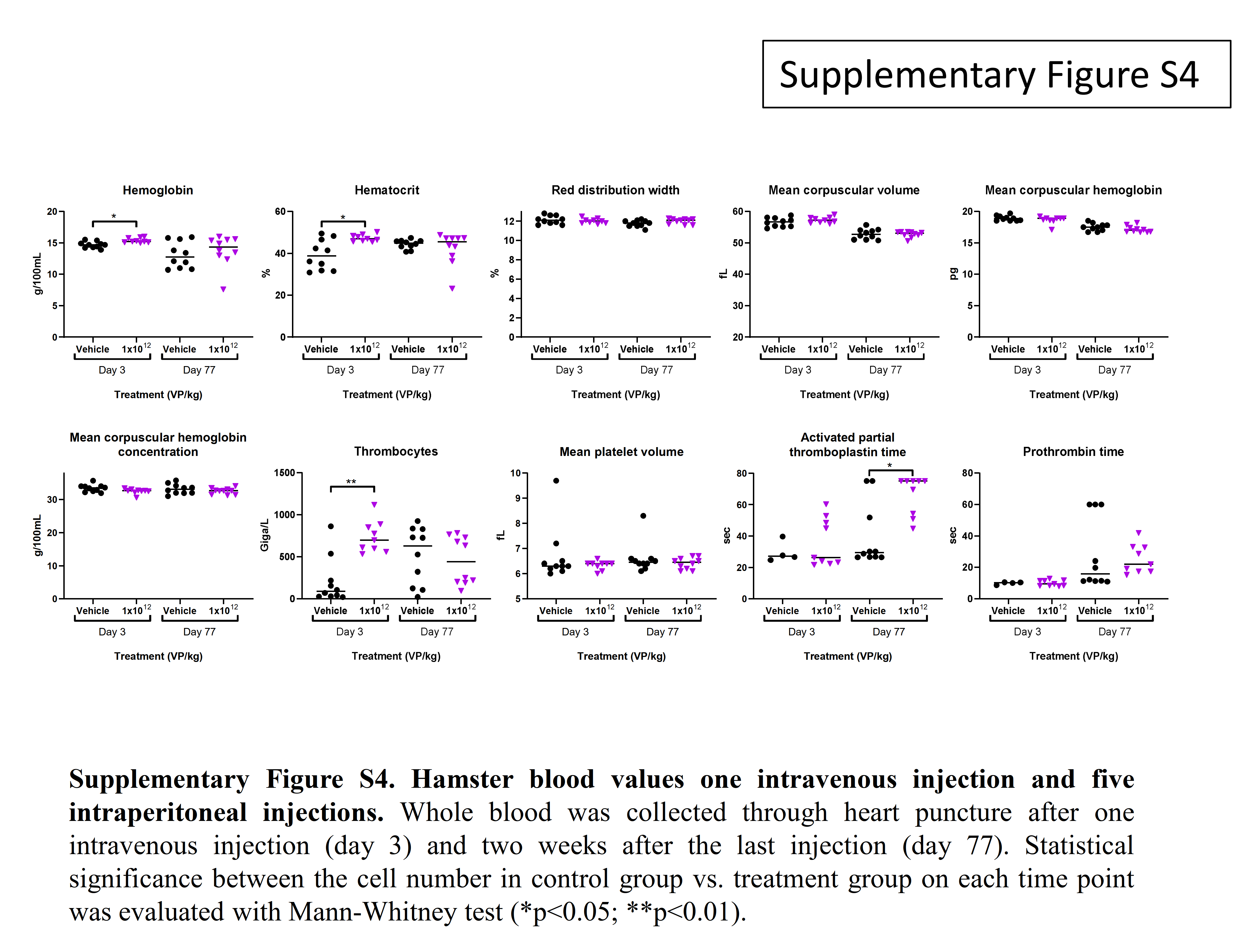

Supplement: Supplementary file 1 [file cells-10-00246-s001.zip › Supplementary figures Havunen 2020/Havunen 2020 Figure_S4.tif]

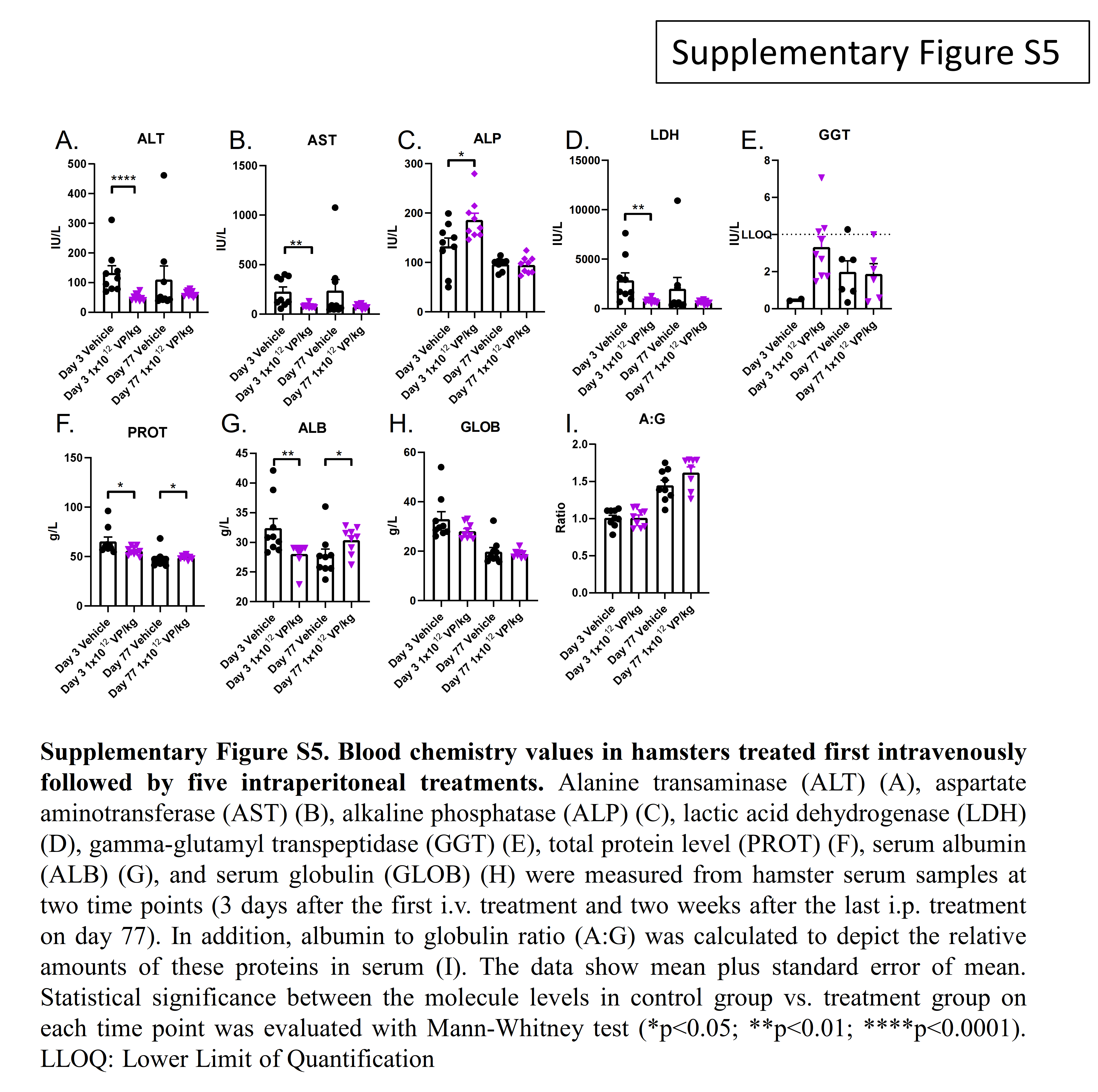

Supplement: Supplementary file 1 [file cells-10-00246-s001.zip › Supplementary figures Havunen 2020/Havunen 2020 Figure_S5.tif]

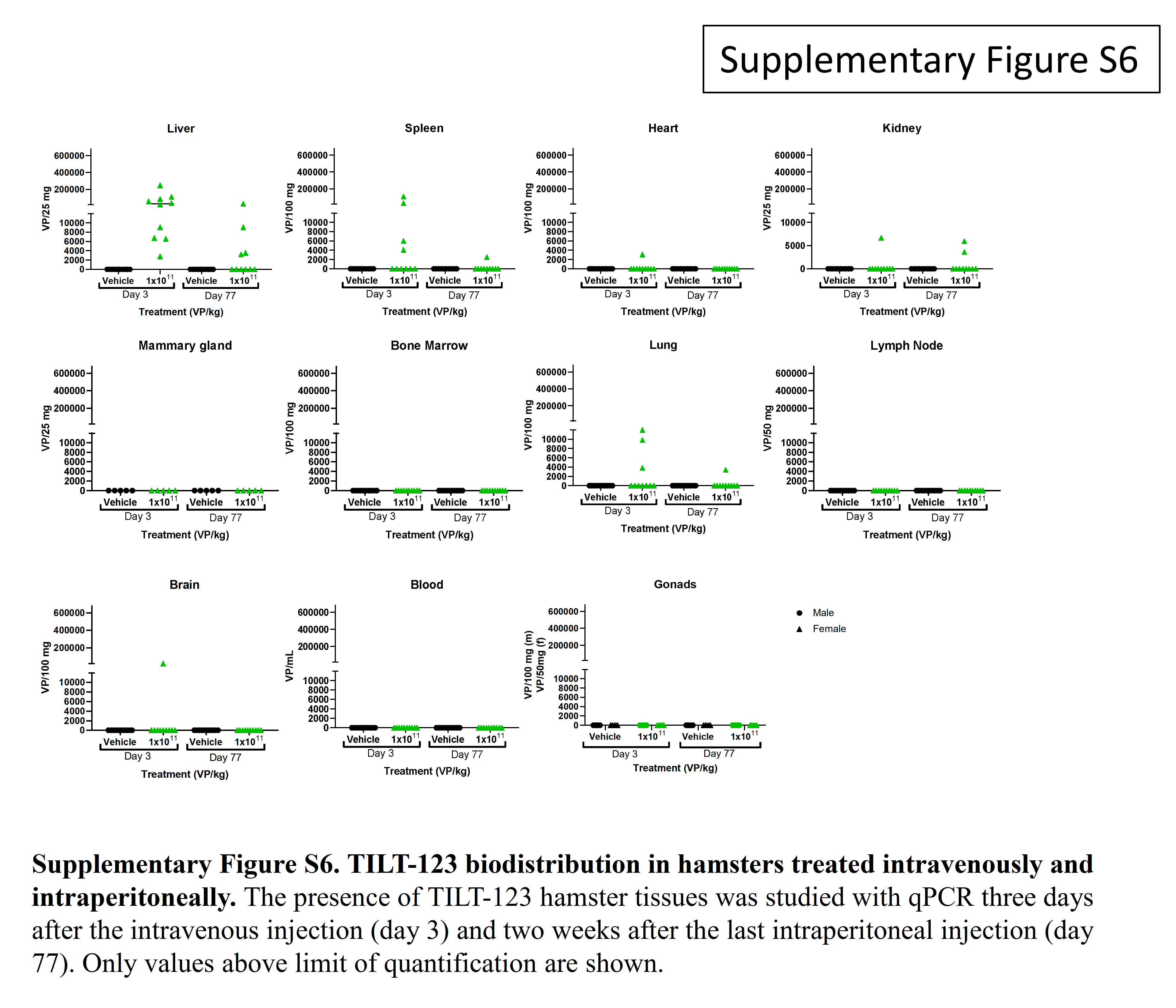

Supplement: Supplementary file 1 [file cells-10-00246-s001.zip › Supplementary figures Havunen 2020/Havunen 2020 Figure_S6.tif]

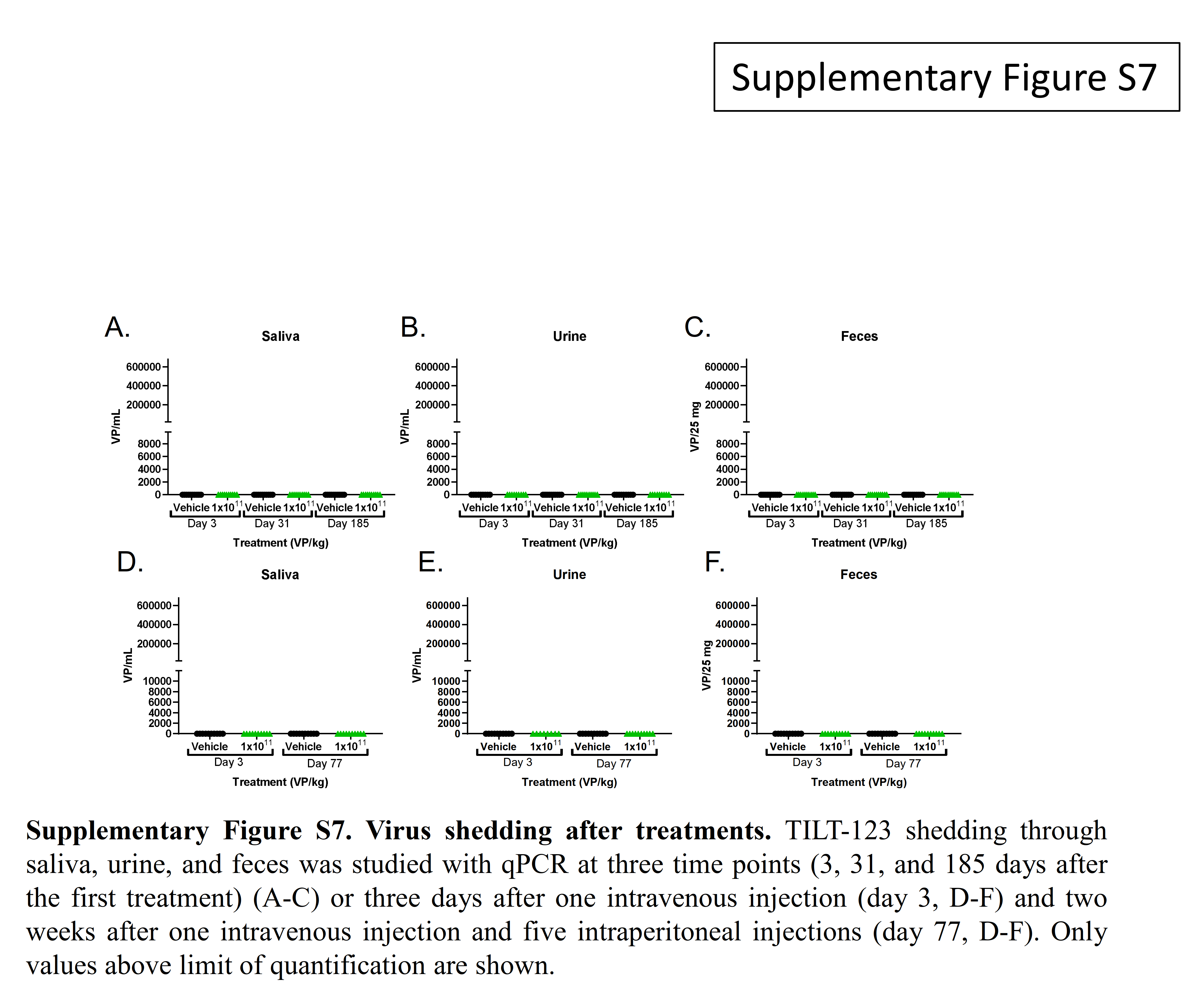

Supplement: Supplementary file 1 [file cells-10-00246-s001.zip › Supplementary figures Havunen 2020/Havunen 2020 Figure_S7.tif]
